# Scoring model to predict risk of chronic kidney disease in Chinese health screening examinees with type 2 diabetes

**DOI:** 10.1007/s11255-021-03045-9

**Published:** 2021-11-01

**Authors:** Xia Cao, Binfang Yang, Jiansong Zhou

**Affiliations:** 1 Department of Health Management, Health Management Research Center of Central South University, The Third Xiangya Hospital, Central South University, Changsha, 410013 Hunan Province China; 2 Department of Psychiatry & Mental Health Institute, The Second Xiangya Hospital, Central South University, Changsha, Hunan Province China

**Keywords:** Chronic kidney disease (CKD), Prediction model, Type 2 diabetes, Risk factors

## Abstract

**Purpose:**

As health screening continues to increase in China, there is an opportunity to integrate a large number of demographic as well as subjective and objective clinical data into risk prediction modeling. The aim of this study was to develop and validate a prediction model for chronic kidney disease (CKD) in Chinese health screening examinees with type 2 diabetes mellitus (T2DM).

**Methods:**

We conducted a retrospective cohort study consisting of 2051 Chinese T2DM patients between 35 and 78 years old who were enrolled in the XY3CKD Follow-up Program between 2009 and 2010. All participants were randomly assigned into a derivation set or a validation set at a 2:1 ratio. Cox proportional hazards regression model was selected for the analysis of risk factors for the development of the proposed risk model of CKD. We established a prediction model with a scoring system following the steps proposed by the Framingham Heart Study.

**Results:**

The mean follow-up was 8.52 years, with a total of 315 (23.20%) and 189 (27.27%) incident CKD cases in the derivation set and validation set, respectively. We identified the following risk factors: age, gender, body mass index, duration of type 2 diabetes, variation of fasting blood glucose, stroke, and hypertension. The points were summed to obtain individual scores (from 0 to 15). The areas under the curve of 3-, 5- and 10-year CKD risks were 0.843, 0.799 and 0.780 in the derivation set and 0.871, 0.803 and 0.785 in the validation set, respectively.

**Conclusions:**

The proposed scoring system is a promising tool for further application of assisting Chinese medical staff for early prevention of T2DM complications among health screening examinees.

**Supplementary Information:**

The online version contains supplementary material available at 10.1007/s11255-021-03045-9.

## Introduction

Type 2 diabetes mellitus (T2DM) is a major cause of chronic kidney disease (CKD). With decades of increasing prevalence of T2DM, diabetes-related CKD is more common than glomerulonephritis-related CKD in the general population in China. In 2010, among hospitalized patients, the percentage with CKD related to diabetes was lower than the percentage with CKD related to glomerulonephritis (0.82 vs. 1.01%). In 2015, the percentage of the hospitalized population with CKD related to diabetes and to glomerulonephritis was 1.10 and 0.75%, respectively [1]. However, the traditional and diabetes-related factors for CKD in individuals with T2DM have rarely been investigated in a Chinese population. Most of the studies included traditional risk factors such as age, sex, body mass index (BMI), blood pressure, fasting plasma glucose (FPG), glycated hemoglobin A1c (HbA1c), lipids indicators, and other traditional risk factors, and seldom include diabetes-related indicators (e.g., medication use and variation in glucose)*.* Diabetes is a group of metabolic diseases characterized by hyperglycemia, and it is associated with long-term multisystem impairment and dysfunction in the cardiovascular system, eyes, kidneys, and nerves [2]. Several reviews and meta-analyses have suggested many risk factors for T2DM, including various biomarkers(increased level of alanine aminotransferase, gamma-glutamyl transferase, uric acid and C-reactive protein, and decreased levels of adiponectin and vitamin D), dietary factors (increased consumption of processed meat and sugar-sweetened beverages, decreased intake of whole grains, and low adherence to a healthy dietary pattern), lifestyle factors (decreased physical activity, high sedentary time, and smoking), environment factors (air pollution), psychosocial factors, medical conditions (high blood pressure, gestational diabetes, metabolic syndrome, preterm birth), and genetic factors [3]. A prediction model for CKD in T2DM patients can help to identify individuals requiring close monitoring. In addition, this model can also be used to discriminate high-risk individuals for preventive interventions targeting the reduction of CKD risk in the future.

Recently, several prediction models of CKD have been constructed for the general population [4–6]. Yun et al. conducted a genome-wide association (GWA) study regarding the development of CKD based on two population-based cohorts of Korean Genome Epidemiology Study and identified several loci highly associated with incident CKD, including LMO7DN, AGL, and SLC35A3, etc. [4]. A simplified risk score can predict the development of decreased glomerular filtration rate (GFR) at 10 years in a Thai general population using readily obtainable clinical and laboratory parameters (including age, sex, systolic blood pressure, history of diabetes, and waist circumference) [5]. In the risk models, each variable was assigned points proportional to the product of its regression coefficient from the multiple logistic regression model for decreased GFR. However, these scoring models above did not include diabetes specific factors, such as hypoglycemic agent and glycemic control. Previous studies have established prediction models for CKD in patients with T2DM [7, 8]. One study using routinely available clinical measurements developed and validated a prediction model for CKD progression in patients with T2DM, demonstrating the influence of adverse metabolic profile on CKD progression [7]. Another study revealed albuminuria and eGFR were the most important factors to predict onset and progression of early CKD in individuals with T2DM [8]. However, these studies did not take the fluctuations of plasma glucose into account. Previous studies demonstrated that glycemic variability was related to diabetic retinopathy, peripheral neuropathy, nephropathy, vascular outcomes [9] and cognitive function [10–12]. Therefore, glycemic variation might be a predictive factor for CKD risk in patients with T2DM. So far, however, there have been no studies establishing such a prediction model in a Chinese mainland population, particularly in the health screening population. A prior retrospective cohort study developed a risk-scoring system for end-stage renal disease in patients with T2DM including variation in HbA1c and variation in systolic blood pressure in Taiwan [13]. In this study, we aimed to develop and validate a prediction model for CKD in Chinese health screening examinees with T2DM.

## Materials and methods

### Study setting and data source

The retrospective cohort study was conducted in Changsha City, capital city of central China' s Hunan Province with a population of 7.1 million (local municipal bureau of statistics, 2019). The recruitment of participants and data collection took place at the Health Management Center of the Third Xiangya Hospital in Changsha between 2009 and 2010. The cohort study database includes information of follow-up visits for health screening examinees, annual health screening records and self-reported questionnaire information. In addition, the study database includes the standardized follow-up records, including the details of revisit records and outpatient and inpatient records to obtain subsequent CKD events one year after the index date to 2018.

### Participants

**Fig. 1:**
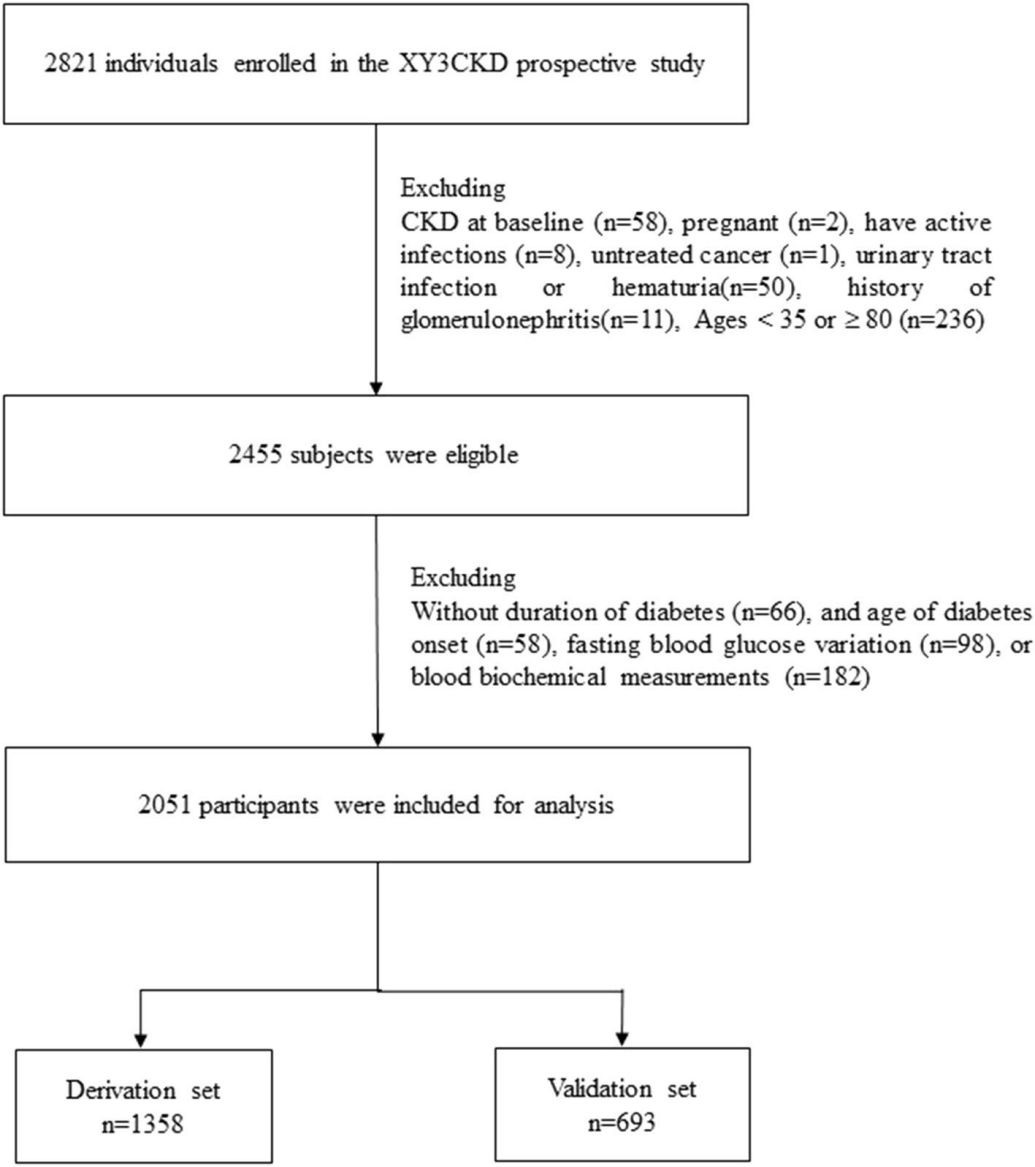
Flowchart of the recruitment procedures for the predictive model of CKD

The study participants consisted of 2821 individuals who were diagnosed with T2DM during 2009–2010 and enrolled in the XY3CKD Follow-up Program. Diabetes was defined as FBG ≥ 7.0 mmol/l, or self-report of physician-diagnosed diabetes or use of anti-diabetes agents. An entry date to the XY3CKD cohort was defined as the index date. XY3CKD, a CKD monitoring program, was established by the Health Management Center of the Third Xiangya Hospital in 2007 [14]. We included patients who had at least one annual follow-up for calculation of visit-to-visit variation in fasting plasma glucose (FPG) and without CKD at baseline or missing data regarding baseline characteristics of medical history and laboratory test results. Patients were excluded if they were less than 35 years old, pregnant, had active infections, untreated cancer and autoimmune disease, or involvement of other suspected causes of renal diseases (e.g., urinary tract infection, polycystic kidney disease, hematuria or history of glomerulonephritis). Figure 1 shows the flowchart for participants in the study. Baseline characteristics were compared between participants included and those excluded using standardized mean differences (Supplementary Table 1). Most of the standardized mean differences were less than 0.10 standard deviations (SD), indicating a negligible difference in proportions or means between included and excluded participants. A total of 2051 subjects were included in the data analysis. All recruited subjects were followed up from the date of cohort entry until either death, incident CKD or withdrawal from the study. The research protocol and procedures were approved by the Research Ethics Board (2016-S077, approved in May 2007) at the Third Xiangya Hospital, Central South University.

### Ascertainment of covariates and outcomes

An interview and a comprehensive assessment of risk factors, status of disease, and complications, were performed for each subject upon enrollment in this study. Blood samples and first-void urine samples were collected between 08:00 and 10:00 a.m. after fasting overnight. Using standard hospital assays, the relevant blood biochemical indexes were measured.

The sociodemographic factors, diabetes-related factors and biomarkers included age, gender, smoking habits, alcohol drinking, physical activity, body mass index, obesity, age of diabetes onset, duration of diabetes, blood pressure, FPG (3.89–6.10 mmol/L), total cholesterol (TC) (2.85–5.69 mmol/L), triglycerides (TG) (0.45–1.69 mmol/L), low-density lipoprotein (2.10–3.12 mmol/L), HDL (1.04–1.96 mmol/L), serum uric acid (male: 149–416 μmol/L; female: 89–357 μmo1/L), creatinine (male: 44–133 μmol/L; female: 70–106 μmo1/L), estimated glomerular filtration rate (eGFR), and the coefficient of variation of fasting plasma glucose (FPG-CV). Current smokers were those who had smoked within 1 year of the survey date and physical inactivity was defined as leisure time activity less than 4 h weekly and predominantly sedentary work. Current drinkers were those who had drunk alcohol at least 12 times but with an average daily consumption of alcohol ≤ 20 g during the past year. Seated blood pressure was measured by skilled, trained nurses after subjects had rested for 15 min. The average of 3 readings was recorded. The formula for BMI was weight (kg) divided by height squared (m^2^) by measurement. Individuals were categorized as normal weight (18.5–23.9 kg/m^2^), overweight (24–27.9 kg/m^2^), and obese (≥ 28 kg/m^2^) according to the Chinese standard [15].

Variation in FPG was measured from health screening or outpatient visits within the first year of the index date for each participant having at least two FPG records in the first year. For each participant, the intrapersonal mean and standard deviation (SD) of all recorded FPG measurements were calculated. The coefficient of variation (CV) was defined as the ratio of the SD over the mean FPG [16]. The CV of FPG was divided by the square root of the ratio of total visits divided by total visits minus 1 to adjust for the possibility that the number of visits might have an effect on the variation. For predictive model development, it was classified into categories based on the tertiles.

Information on each specific comorbidity including hypertension (ICD-9-CM codes 404–405), stroke (ICD-9-CM codes 431–438), ischemic heart disease (ICD-9-CM codes 410–414), carotid atherosclerosis (ICD-9-CM codes 440), diabetes retinopathy (ICD-9-CM codes 362.0), hyperlipidemia (ICD-9-CM codes 272), and hyperuricemia (ICD-9-CM codes 274 and 790.6) were identified. The carotid ultrasound examinations were performed blinded by six experienced sonographers with an ultrasound B-mode system equipped with a 7.5 MHz linear array probe. The sonographers must have more than 3-year experience in carotid sonography following a standard carotid ultrasound research protocol. Carotid intima-medial thickness (cIMT) was calculated as the mean of cIMT of the far walls of both common carotid arteries of both carotid bulbs as described elsewhere [17]. The upper quartile of cIMT (≥ 0.7 mm) was defined as increased cIMT and plaque was defined as a focal wall thickening or protrusion in the lumen > 50% of the surrounding thickness [18]. Hyperuricemia was defined as serum uric acid concentrations ≥ 420 µmol/l in men and ≥ 360 µmol/l in women [19]. In addition, relevant information on antihypertensive medication, antidiabetic treatment, and lipid-lowering therapy was also collected.

### Renal outcome definitions

Serum creatinine (Scr) was measured using an enzymatic method. The modified Chinese equation was used to calculate estimated glomerular filtration rate (eGFR) as follows: eGFR (mL/min/1.73 m^2^) = 175 × (Scr in enzymatic method)^−1.234^ × age ^− 0.287^ (× 0.79, if female) [20]. Proteinuria was diagnosed using a urine dipstick test and was considered positive for a result of ≥ 1 + , corresponding to a urinary protein level > 30 mg/dL [21]. CKD was defined as positive proteinuria and/or eGFR < 60 mL/min/1.73 m^2^ [6]. If a participant during follow-up experienced more than one CKD event, only the first outcome contributed to the final analysis. The date of onset of CKD was defined as the midpoint between the last visit when the participant did not have CKD and the first visit when the participant was diagnosed with CKD. The follow-up period was calculated as the number of days from the date of observation to the date of CKD diagnosis or to the date of the final visit.

### Statistical analyses

Continuous variables were presented as means with standard deviations and categorical variables were presented as frequency and proportions. Cohen’s d test was used to assess the effect size of the standardized difference between derivation and validation sets. Cox proportional hazards models were used to calculate crude and multivariate-adjusted hazard ratios with 95% confidence interval for predictors of CKD. A multivariable Cox model was developed using the backward elimination approach with candidate predictors. All eligible study subjects were randomly divided into a derivation group and a validation group at a 2:1 ratio. The derivation set was applied to generate a prediction model, and the validation set was applied to assess the predictive accuracy and calibration. Any variable with a significant univariate test of a *P* value < 0.20 was selected as a candidate for multivariable analysis [22]. Then a multivariable model with candidate variables with *P* value < 0.05 without collinearity was constructed. The assumption of Cox’s proportional hazards was assessed for all variables in our multivariate model, after refining a main effects model. The incidence rates of CKD were evaluated by calculating the incidence rate per 1000 person-years applying a formula as follows: incidence rate = number of incident cases/person-years × 1000.

The steps for predictive model development were based on the Framingham Heart study to determine the CKD risk score [23]. The construction steps were as follows: (1) we estimated the parameters of the multivariate Cox’s proportional hazards model with the approach mentioned above for model building strategy; (2) the risk factors were classified into categories and their reference values Wij were determined; (3) we assigned a score for each category to determine the referent risk factor profile with a base category 0 score; (4) we determined the distance from the base category to each class in regression units; (5) we set the constant B which was the number of regression units reflecting one point in the final points system; (6) we calculated the number of points for each category of each risk factor, where Point_ij_ = (W_ij_ − W_iREF_)/β; (7) we determined the prediction risks for all possible total scores by the following equation: $\widehat{p}=1-S$_0_(t)^exp (^$\Sigma \beta i\times Xi-\beta i\times \widehat{X}i)$, where $\widehat{p}$ is the baseline disease free probability, β_i_ is the regression coefficient for X_i_, and the $\widehat{X}i$ is the mean level of X_i_. The constant β is determined by the regression coefficient of age in the multivariate model. The receiver operating characteristic (ROC) curve analysis was applied to assess the predictive accuracy, and area under the curve (AUC) was used to assess the discriminatory ability of the predictive model. AUC values vary between 0 and 1, with a value over 0.7 representing a good discriminatory ability. The Hosmer–Lemeshow χ^2^ test evaluated goodness of fit by comparing the observed versus the predicted events. Statistical analyses were conducted using SAS Version 9.3 (SAS Institute, Cary, NC, USA) and MedCalc 19.0 (MedCalc Software, Ostend, Belgium). The significance level was set at *P* < 0.05 (two tailed).

## Results

This retrospective cohort study included 2051 patients with T2DM who were free of CKD at baseline and aged 35–78 years. During a mean follow-up of 8.52 years, 315 (23.20%) and 189 (27.27%) newly diagnosed CKD cases were identified in the derivation and validation sets, respectively (Table 1). As shown in Table 1, the standardized effect sizes of each variable were all less than 0.20, representing that all baseline variables were comparable between the two sets.

**Table1:** Baseline characteristics of participants

Variables*	Derivation set (*n* = 1358)	Validation set (*n* = 693)	Standardized effect size	*P* value
Socio-demographic factors				
Age (years)	56.44 ± 10.43	59.89 ± 9.78	− 0.17	< 0.01
Sex				
Female	315(23.20)	154 (22.22)	− 0.01	0.62
Male	1043(76.80)	539(77.78)		
Smoking habit	390(28.79)	191(27.56)	− 0.01	0.58
Alcohol drinking	154(11.34)	91(13.13)	0.03	0.24
Physical inactivity	567(41.75)	259(37.37)	− 0.04	0.06
Body mass index (kg/m^2^)	25.38 ± 3.04	25.07 ± 2.46	0.11	0.02
Obesity (BMI ≥ 28 kg/m^2^)	231(17.01)	63(9.09)	− 0.11	< 0.01
Diabetes-related factor and biomarker				
Age of diabetes onset (years)	52.38 ± 9.67	52.25 ± 9.62	0.01	0.98
Duration of type 2 diabetes (years)	4.67 ± 4.19	5.31 ± 4.52	0.01	< 0.01
Systolic blood pressure (mmHg)	136.28 ± 19.66	137.74 ± 19.71	− 0.07	0.11
Diastolic blood pressure (mmHg)	81.34 ± 12.51	80.63 ± 9.66	0.03	0.19
Fasting blood glucose (mmol/l)	7.57 ± 2.46	7.68 ± 2.31	− 0.02	0.36
Total cholesterol (mmol/l)	4.94 ± 0.92	5.14 ± 1.16	− 0.09	< 0.01
Triglyceride (mmol/l)	2.53 ± 2.36	2.81 ± 2.31	− 0.06	0.65
Low-density lipoprotein (mmol/l)	2.81 ± 0.86	2.95 ± 0.81	− 0.08	0.03
High-density lipoprotein (mmol/l)	1.17 ± 0.29	1.22 ± 0.29	− 0.09	< 0.01
Serum uric acid(µmol/l)	306.54 ± 85.27	327.33 ± 79.31	− 0.17	< 0.01
Creatinine (µmol/l)	69.16 ± 13.14	70.45 ± 11.90	− 0.04	0.03
eGFR (mL/min/1.73 m^2^)	114.22 ± 24.27	109.93 ± 21.21	0.09	< 0.01
Variation of fasting blood glucose (%)	17.06 ± 11.04	18.42 ± 9.91	− 0.06	< 0.01
Comorbidity				
Hypertension	490 (36.08)	252(36.36)	0.003	0.90
Stroke	14 (1.03)	7(1.01)	− 0.001	0.97
Coronary artery disease	203 (14.95)	112(16.16)	0.01	0.68
Carotid atherosclerosis	97 (7.14)	53(7.65)	0.01	0.68
Diabetes retinopathy	42 (3.09)	14(2.02)	− 0.03	0.16
Hyperlipidemia	294 (21.65)	175(25.25)	0.04	0.07
Hyperuricemia	182 (13.40)	84(12.12)	− 0.02	0.41
Medication use				
Anti-diabetes medications				
No medication	499 (36.75)	258(37.23)	0.005	0.83
Oral only	737 (54.27)	374(53.97)	− 0.003	0.90
Insulin	65 (4.79)	34(4.91)	0.003	0.91
Insulin + oral agent	57 (4.20)	27(3.89)	− 0.007	0.75
Hypertension medications	469 (34.54)	266(38.38)	0.04	0.09
Cardiovascular medications	98 (7.22)	28(4.04)	− 0.06	< 0.01
Lipid medications	206 (15.17)	104(15.01)	− 0.002	0.92
Renal outcome				
CKD	315 (23.20)	189(27.27)	0.05	0.04

*BMI* body mass index, *eGFR* estimated glomerular filtration rate

*Mean ± SD or *n* (%)

Analysis by Cox's proportional hazard model indicated that significant factors included age, male, duration of type 2 diabetes, body mass index ≥ 28 kg/m^2^, variation of fasting plasma glucose ≥ 35%, comorbidity with stroke, and comorbidity with hypertension (all *P* < 0.05) (Table 2). The numbers of participants, CKD cases, the cumulative incidence rates, person-years, hazard ratios and* P* values for baseline predictors are presented in Table 2. Regression coefficients of factors retained in the final model are presented in Table 3. The assigned score for each factor was five-fold of the regression coefficient of age, and the risk score was calculated by adding the scores of all factors (Table 3). The calculated risk scores ranged from 0 to 15. The 3-, 5- and 10-year risks of CKD were estimated for each point (Table 4).

**Table 2:** Number of participants, incidence rates of CKD, and crude hazard ratios by baseline CKD predictors

Variables	*N*	Cases	Person-year	Incidence rate	Crude HR (95% CI)	*P*
Socio-demographic factors						
Age (years)	2051	504	17,479	28.83	1.85 (1.12, 3.17)	0.02
Sex						
Female	469	77	4158	18.52	1.00	
Male	1582	427	13,321	32.05	1.33 (1.12, 1.68)	0.04
Diabetes-related factor and biomarker						
Duration of type 2 diabetes (years)						
0	246	22	2096	10.50	1.00	
1–5	964	202	8213	24.60	1.83 (1.49, 2.25)	0.02
6–10	246	62	2096	29.58	2.20 (1.72, 2.93)	< 0.01
11–15	267	82	2275	36.04	2.69 (2.03, 3.56)	< 0.01
16–20	205	66	1747	37.78	2.82 (2.15, 3.72)	< 0.01
≥ 20	123	70	1048	66.79	4.96 (3.87, 6.32)	< 0.01
Body mass index (kg/m^2^)						
< 24	637	102	5427	18.79	1.00	
24–28	1120	255	9542	26.72	1.09 (1.05, 1.39)	0.12
≥ 28	294	147	2505	58.68	1.86 (1.51, 2.36)	0.02
Variation of fasting plasma glucose (%)						
< 17.6	1183	238	10,079	23.61	1.00	
17.6–35.0	707	203	6024	33.70	1.09 (1.05, 1.40)	0.12
≥ 35.0	161	63	1372	45.92	1.49 (1.26, 1.88)	0.02
Comorbidity						
Stroke						
No	2030	490	17,296	28.33	1.00	
Yes	21	14	179	78.21	2.17 (1.66, 2.77)	< 0.01
Hypertension						
No	1309	294	11,153	26.36	1.00	
Yes	742	210	6322	33.22	1.26 (1.23, 1.61)	0.04

*HR* hazard ratio, *CI* confidence intervals

Incidence rate = number of incident cases/person-years × 1000

**Table 3:** CKD risk score sheet based on the final multivariate Cox proportional hazards model

Risk factor	β (SE)	Mean or proportion	*P*	Point
Socio-demographic factors				
Age (years)	0.12 (0.07)	56.44	0.02	0–8
Sex(male)	0.27 (0.12)	0.24	0.03	1
Diabetes-related factor and biomarker				
Duration of type 2 diabetes (years) (reference = 0)				
1–5	0.33 (0.07)	0.47	0.02	1
6–10	0.48 (0.15)	0.12	<0.01	1
11–15	0.56 (0.17)	0.13	<0.01	1
16–20	0.60 (0.20)	0.10	<0.01	1
≥ 20	1.62 (0.46)	0.06	<0.01	2
Body mass index (kg/m^2^) (reference < 24)				
24–28	0.05 (0.11)	0.55	0.12	0
≥ 28	0.35 (0.09)	0.14	0.02	1
Variation of fasting blood glucose (%) (reference < 17.6)				
17.6–35.0	0.08 (0.12)	0.34	0.12	0
≥ 35.0	0.28 (0.05)	0.08	0.02	1
Comorbidity				
Stroke	0.51 (0.15)	0.01	˂0.01	1
Hypertension	0.24 (0.05)	0.36	0.04	1

**Table 4:** 3-, 5-, and 10-year estimated CKD risks of each possible sum of points

Point total	Predicted risk of CKD		
	3-Year risk	5-Year risk	10-Year risk
0	0.0008	0.0014	0.0039
1	0.0012	0.0026	0.0067
2	0.0022	0.0043	0.0115
3	0.0038	0.0075	0.0198
4	0.0066	0.0130	0.0340
5	0.0113	0.0223	0.0579
6	0.0195	0.0382	0.0981
7	0.0332	0.0652	0.1638
8	0.0568	0.1100	0.2674
9	0.0963	0.1829	0.4201
10	0.1609	0.2965	0.6216
11	0.2629	0.4609	0.8408
12	0.4138	0.6702	0.9138
13	0.6139	0.8854	0.9937
14	0.8340	0.9603	1.0000
15	0.9704	1.0000	1.0000

**Fig. 2:**
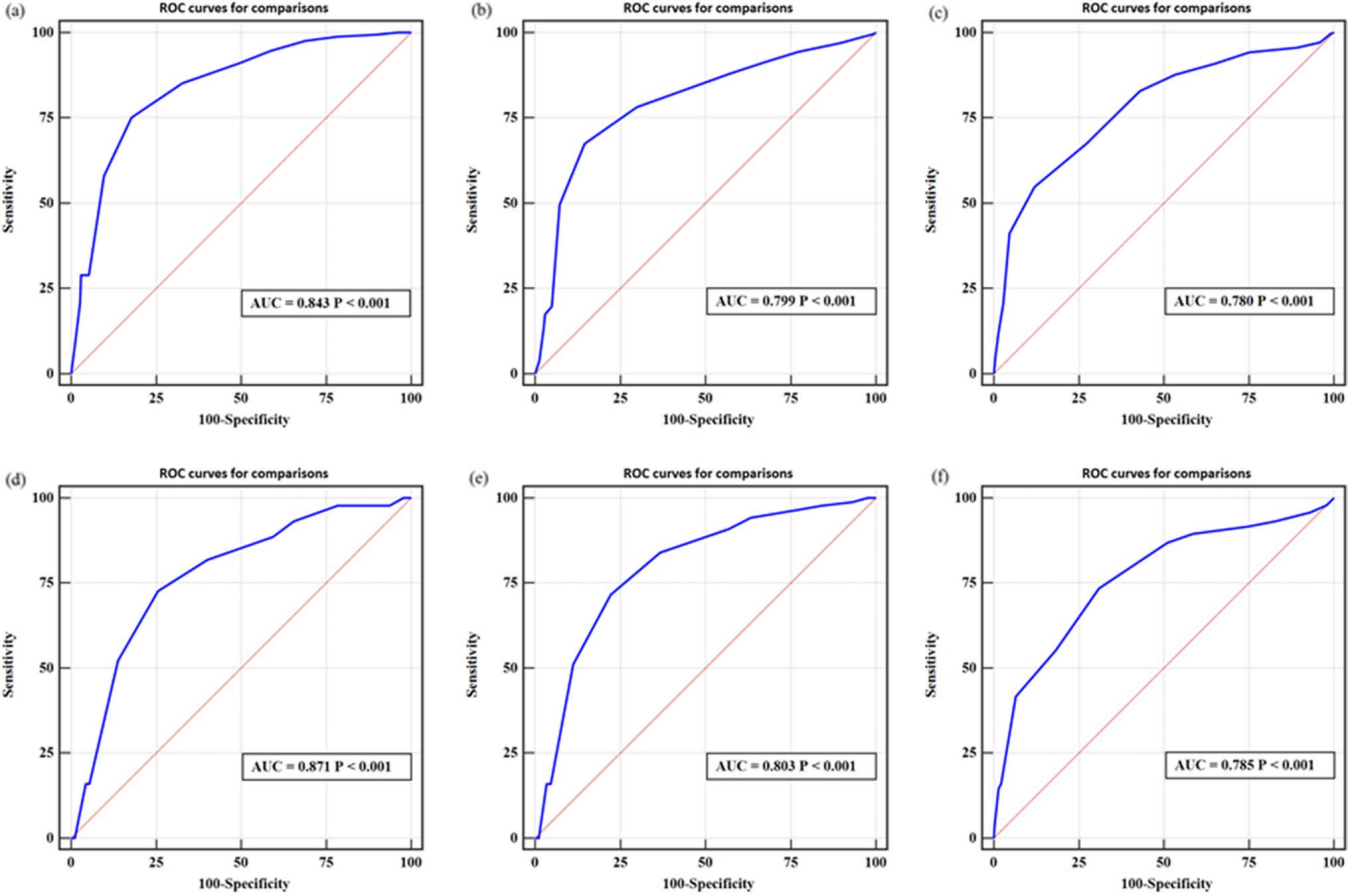
Receiver operating characteristic curve (ROC) for 3-year, 5-year and 10-year CKD risks in the derivation set and in the validation set, respectively

Figure 2 shows the AUCs for 3-, 5- and 10-year CKD risks in the derivation and validation sets. The AUCs and their 95% confidence interval for 3-, 5- and 10-year CKD risks were 0.843 (0.826, 0.858), 0.799 (0.767, 0.828) and 0.780 (0.762, 0.798) in the derivation set and 0.871 (0.846, 0.886), 0.803 (0.785, 0.841) and 0.785 (0.772, 0.803) in the validation set, respectively, demonstrating that our prediction model displayed good discrimination ability.

**Fig. 3:**
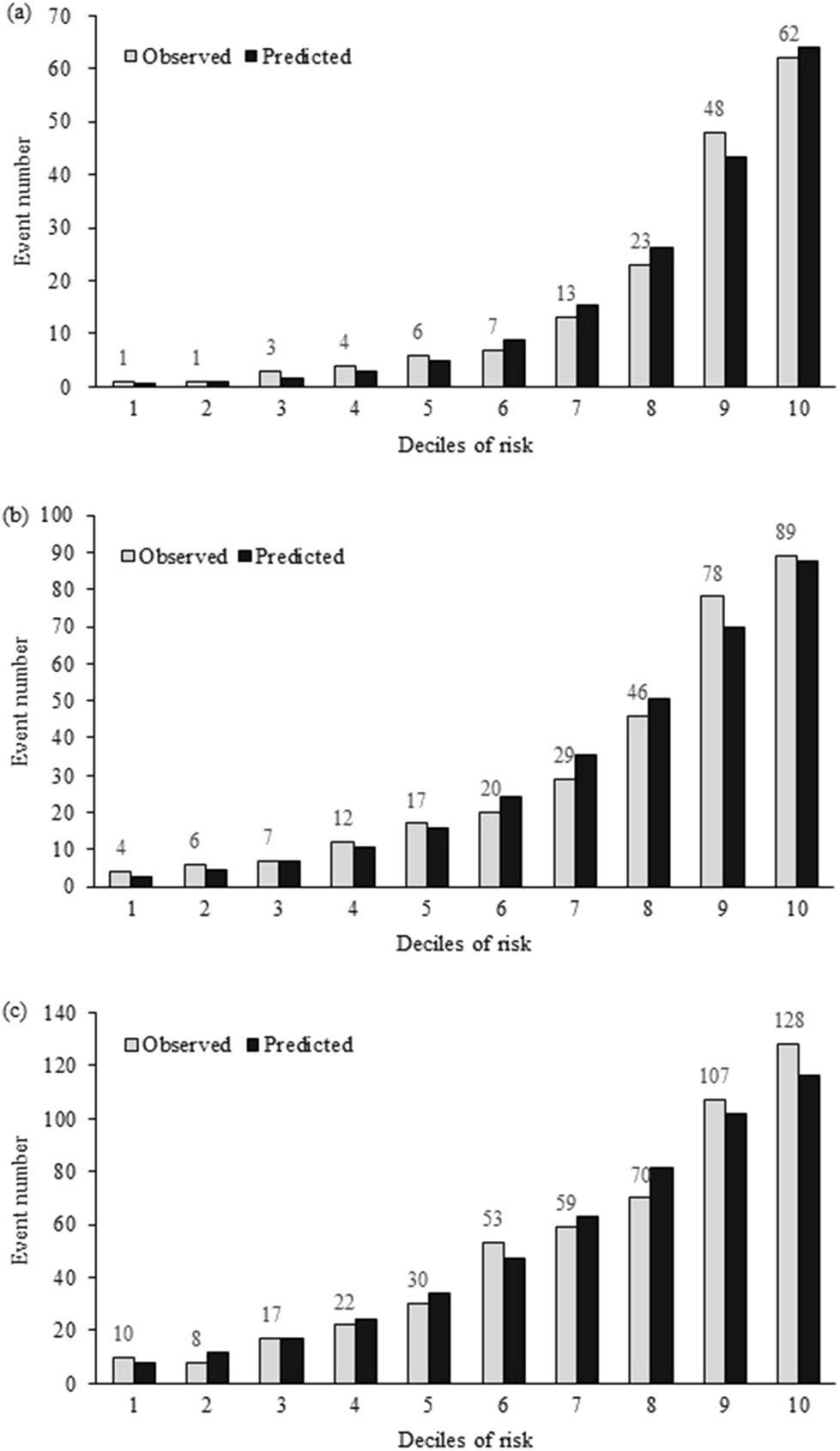
Predicted versus observed CKD numbers according to deciles of 3-year, 5-year and 10-year CKD risks in the validation set

Figure 3 presents the calibration plots comparing actual and predicted CKD events by deciles of 3-, 5- and 10-year risks in the derivation and validation sets. The results of the Hosmer–Lemeshow χ^2^ test for 3-, 5- and 10-year risks in the validation set demonstrated excellent goodness of fit.

## Discussion

In this retrospective cohort study of patients with T2DM, more than one-fourth developed CKD in nearly 9 years of follow-up. To our knowledge, the proposed model is the first established model to predict CKD risk, specifically for Chinese health screening examinees with T2DM. We identified several significant independent risk factors in our derivation cohort, including age, sex, duration of T2DM, BMI, variation in FPG, stroke, and hypertension. These risk factors reflect both the duration and intensity of diabetic pathology and its vascular complications. We established a CKD risk prediction model, which demonstrated good predictive power for CKD risk, with reference to risk score function proposed by the Framingham Heart Study. The prediction model for CKD showed good discrimination ability over 3-, 5- and 10-year periods both in the derivation and validation sets, with AUCs of 0.843, 0.799, and 0.780 for the derivation set, respectively, and 0.871, 0.803, and 0.785 in the validation set, respectively.

Several CKD prediction models have been developed for the general population [4–8] and two models were developed for T2DM patients [7, 8]. The predictive model for CKD progression in T2DM in Singapore is based on a prospective study with median follow-up of 5.5 years [7]. The two prediction models both used easily available demographic and biomarker variables, but without glycemic variability. However, our prediction model involved the variation in FPG. Xiao et al. developed and compared several predictive models using statistical, machine learning and neural network approaches [24]. Features in routine blood tests, including albumin, Scr, TG, LDL and eGFR levels, showed predictive ability for CKD severity. Another CKD prediction was conducted in Asian subjects considering similar factors to the prior study [7]. SBP and BMI are two common factors in these two studies conducted in the general population. Similarly, our prediction model reveals that BMI ≥ 28 kg/m^2^ is associated with increased risk of CKD. Previous studies including our study have suggested that obesity increases the risk of CKD [14, 25–27]. High blood pressure may increase pressure load in the kidney, which may cause kidney injury. Previous literature reports that high blood pressure is associated with increased CKD risk [28–30]. In addition, Laible et al. reports renal dysfunction is far more common in stroke patients than in the general population [31]. Similar results are observed in our study. We found that comorbidity with hypertension or history of stroke was significant predictor of CKD risk.

Glycemic variability, an index of glucose control known as blood glucose fluctuation, is closely associated with decreased eGFR and an increased risk of CKD in T2DM patients with poor glycemic control [32]. Costantino et al. presented that glucose fluctuations contribute to chromatin remodeling and oxidative stress and may explain persistent vascular dysfunction in patients with T2DM patients [33]. The present prediction model found that variation in FPG was a significant factor for CKD, which was not included in prior similar models. In recent years, increasing evidence suggests that one common pathological mechanism shared in early stages of diabetes-related CKD is microvascular endothelial dysfunction leading to the development of albuminuria and decreased GFR during the disease process [34–36]. Our results suggest that variability in glucose is a predictor of CKD and the underlying mechanisms may be microvascular endothelial dysfunction, leading to renal impairment.

The predictive model in Singapore showed similar levels of discrimination (the AUCs for the development and validation datasets were 0.80 and 0.83, respectively) [7]. Despite slightly younger T2DM patients (mean age 56.4 years old vs. 57.3 years old), our results present a higher AUC value of 0.84 and 0.87 in the development and validation sets, respectively. Beyond that, with increasing incidence of CKD cases among patients younger than 40 years old, the present model shows the capability of external generalization. In the present study, 14.29 and 14.78% of incident cases aged 40 years and younger were identified in both the derivation and validation cohorts. Of course, the risk of CKD in patients 40 years and older should be more closely monitored. Hosmer–Lemeshow test results revealed that the model had good predictive ability in the validation set. AUCs for 3-, 5- and 10-year periods in the sensitivity analysis were 0.871, 0.803 and 0.785, respectively, demonstrating that our results are robust.

The strengths of our study include a relatively adequate sample size of T2DM patients, a sufficiently long observation period, and inclusion of diabetes indicators and novel predictors of glucose variation. However, some limitations of our study must be considered. First, the exposures at baseline may vary, and the time variations could not be considered in the present study, which may have resulted in selection bias and affected model accuracy. Although the differences in some baseline variables were statistically significant, the actual differences were clinically quite small for most variables (all standardized effect size values less than 0.5). Second, the cohort database did not contain information about genetic factor or variation of hemoglobin A1c. Third, our study took place in a single center from a large urban teaching hospital, and the study populations being the health screening examinees could lead to selection bias. Fourth, the diagnosis of CKD in the present study depended on the value of creatinine or positive proteinuria on only one occasion and is accordingly more prone to misclassification.

The current study has several clinical implications. The developed scoring model, based on the XY3CKD cohort, may facilitate more appropriate clinical decision-making for clinicians and patients than that provided by the CKD stages recommended in the existing clinical guidelines, which are based on eGFR and proteinuria (albuminuria) alone [37]. Applying the scoring model developed and validated in the present study could also help to provide individual diabetes-related CKD patients with the necessary knowledge and interventions at the optimal time. With the information technology integrating data modeling and electronic medical record for health care has been applied in clinical settings, a risk score calculator consisting of easily available variables and dynamic variables such as variation of FPG become feasible. The risk score calculator can be integrated into the information system of primary health care for monitoring and intervention.

## Conclusion

In conclusion, this study demonstrated a risk scoring system for predicting CKD in Chinese health screening examinees with type 2 diabetes. This developed prediction model for 3-, 5- and 10-year CKD risks demonstrated good prediction accuracy and discriminatory ability. The system may be used in clinical settings to provide a simple tool for clinicians, policy-makers and patients.

## Supplementary Information

Below is the link to the electronic supplementary material.

Supplementary file1 (DOCX 28 KB)
