# Supplementary material for: Scoring model to predict risk of chronic kidney disease in Chinese health screening examinees with type 2 diabetes
Source: Int Urol Nephrol. 2021 Nov 1;54(7):1629–39. doi: 10.1007/s11255-021-03045-9 (PMC9184348; doi:10.1007/s11255-021-03045-9)
Supplement: Supplementary file 1 — Supplementary file1 (DOCX 28 KB) [file 11255_2021_3045_MOESM1_ESM.docx]

**Revised Supplementary Table1. Baseline characteristics of the study population in excluded group and included group**

| Variables* | Excluded group  （n=404） | Included group  （n=2051） | Standardized effect size | *P* value |
| --- | --- | --- | --- | --- |
| ***Socio-demographic factors*** | | | |  |
| Age (years) | 58.14±10.72 | 57.59±10.31 | 0.05 | 0.35 |
| Sex |  |  | 0.001 | 0.95 |
| Female | 93(23.02) | 469 (22.87) |  |  |
| Male | 311(76.98) | 1582(77.13) |  |  |
| Smoking habit | 117(28.96) | 581(28.33) | 0.35 | ˂0.01 |
| Alcohol drinking | 42(10.40) | 245(11.95) | -0.02 | 0.38 |
| Physical inactivity | 169(41.83) | 826(40.27) | 0.01 | 0.56 |
| Body mass index (kg/m^2^) | 25.16±2.96 | 25.28±2.85 | -0.04 | 0.77 |
| Obesity (BMI≥28 kg/m^2^) | 53(13.12) | 301(14.68) | -0.02 | 0.42 |
| ***Diabetes-related factor and biomarker*** | | | |  |
| Age of diabetes onset (years) | 52.58±9.82 | 52.31±9.64 | 0.01 | 0.14 |
| Duration of type 2 diabetes (years) | 5.22±4.48 | 5.34±4.59 | -0.01 | 0.62 |
| Systolic blood pressure (mmHg) | 137.56±19.58 | 136.76±19.63 | 0.02 | 0.97 |
| Diastolic blood pressure (mmHg) | 81.05±11.26 | 81.11±11.61 | -0.01 | 0.11 |
| Fasting blood glucose (mmol/l) | 7.95±2.66 | 7.61±2.41 | 0.07 | ˂0.01 |
| Total cholesterol (mmol/l) | 5.12±1.07 | 5.00±1.01 | 0.06 | ˂0.01 |
| Triglyceride (mmol/l) | 2.48±2.68 | 2.34±2.62 | 0.03 | 0.04 |
| Low-density lipoprotein (mmol/l) | 2.87±0.86 | 2.85±0.85 | 0.01 | 0.83 |
| High-density lipoprotein (mmol/l) | 1.18±0.27 | 1.19±0.29 | -0.02 | 0.27 |
| Serum uric acid(µmol/l) | 318.96±84.35 | 313.49±83.65 | 0.03 | 0.54 |
| Creatinine (µmol/l) | 69.85±12.82 | 69.59±12.72 | 0.01 | 0.70 |
| eGFR (mL/min/1.73 m^2^) | 115.62±24.16 | 112.79±23.31 | 0.06 | ˂0.01 |
| Variation of fasting blood glucose (%) | 18.17±11.12 | 17.51±10.66 | 0.03 | 0.15 |
| ***Comorbidity*** | | | |  |
| Hypertension | 142(35.15) | 742(36.18) | -0.01 | 0.69 |
| Stroke | 5(1.24) | 21(1.02) | 0.01 | 0.70 |
| Coronary artery disease | 60(14.85) | 315(15.36) | -0.01 | 0.80 |
| Carotid atherosclerosis | 30(7.43) | 150(7.31) | 0.002 | 0.94 |
| Diabetes retinopathy | 12(2.97) | 56(2.73) | 0.005 | 0.79 |
| Hyperlipidemia | 85(21.04) | 469(22.87) | -0.02 | 0.42 |
| Hyperuricemia | 51(12.62) | 266(12.97) | -0.004 | 0.85 |
| ***Medication use*** | | | |  |
| Anti-diabetes medications |  |  |  |  |
| No medication | 138(34.16) | 757(36.91) | -0.02 | 0.29 |
| Oral only | 223(55.20) | 1111(54.17) | 0.47 | ˂0.01 |
| Insulin | 22(5.45) | 99(4.83) | 0.01 | 0.60 |
| Insulin + oral agent | 21(5.20) | 84(4.10) | 0.02 | 0.34 |
| Hypertension medications | 152(37.62) | 735(35.84) | 0.01 | 0.49 |
| Cardiovascular medications | 26(6.44) | 126(6.14) | 0.004 | 0.82 |
| Lipid medications | 55(13.61) | 310(15.11) | -0.48 | ˂0.01 |
| ***Renal outcome*** |  |  |  |  |
| CKD | 102(25.25) | 504(24.57) | 0.01 | 0.77 |

*: Mean ± SD or n (%)

Abbreviations: BMI, body mass index; eGFR, estimated glomerular filtration rate.
